# The predictive role of soluble programmed death ligand 1 in digestive system cancers

**DOI:** 10.3389/fonc.2023.1170220

**Published:** 2023-07-13

**Authors:** Jian Ruan, Zhihong Zhao, Yuting Qian, Ruilian Xu, Guixiang Liao, Feng-Ming (Spring) Kong

**Affiliations:** ^1^ The Second Clinical Medical College, Jinan University, Guangdong, China; ^2^ Department of Nephrology, Shenzhen People’s Hospital, The Second Clinical Medical College, Jinan University, Guangdong, China; ^3^ Department of Clinical Oncology, Hong Kong University Shenzhen Hospital and Queen Mary Hospital, Hong Kong University Li Ka Shing Medical School, Hong Kong, Hong Kong SAR, China; ^4^ Department of Clinical Oncology, Queen Mary Hospital, Hong Kong University Li Ka Shing Faculty of Medicine, Hong Kong, Hong Kong SR, China

**Keywords:** digestive system cancers, overall survival, prognosis, soluble programmed death ligand 1, gastric cancer

## Abstract

**Introduction:**

The prognostic role of soluble programmed death ligand 1 (sPD-L1) in digestive system cancers (DSCs) remains inconclusive. This study aimed to explore the predictive value of sPD-L1 expression in DSCs.

**Methods:**

Comprehensive searches were run on the electronic databases (PubMed, Web of Science, EMBASE, and the Cochrane Library) to identify studies that assessed the prognostic role of sPD-L1 in DSCs. Review Manager software (version 5.3) was used for all analyses. Pooled data for survival outcomes were measured as hazard ratios (HRs), 95% confidence intervals (CIs), and odds ratios and their 95% CIs.

**Results:**

The search identified 18 studies involving 2,070 patients with DSCs. The meta-outcome revealed that a high level of sPD-L1 was related to poorer overall survival (HR, 3.06; 95% CI: 2.22–4.22, p<0.001) and disease-free survival (HR, 2.53; 95% CI: 1.67–3.83, p<0.001) in DSCs. Individually, the prognostic significance of high level of sPD-L1 expression was the highest in hepatic cell carcinoma (HR, 4.76; p<0.001) followed by gastric cancer (HR=3.55, p<0.001).

**Conclusion:**

sPD-L1 may be a prognostic factor in DSCs for overall survival and disease-free survival. Inflammatory cytokines, treatment approaches, and other factors may affect the expression of sPD-L1. Therefore, the prognostic value of sPD-L1 for recurrence and metastasis should be further investigated. sPD-L1 may also predict response to treatment. Well-designed prospective studies with standard assessment methods should be conducted to determine the prognostic value of sPD-L1 in DSCs.

## 1 Introduction

Digestive system cancer (DSC) is a common malignant neoplasm (1). DSCs include cancers of the intestines, pancreas, esophagus, stomach, and liver (2). Colorectal cancer (CRC) is the third most common cancer, and 1.9 million new CRC cases and 935,000 deaths (3) were reported in 2020 (4). The treatment of CRC includes surgery, chemotherapy, radiotherapy, targeted therapy, and immunotherapy (5). Approximately half of CRC patients with a resectable primary tumor will subsequently develop metastatic disease (6). Patients of stage IV CRC is with only 11% survival rate (7). Pancreatic cancer is with poor survival (4). Moreover, pancreatic cancer is projected to be the second leading cause of cancer-related deaths by 2030 (8). Operative way is the standard approach for resectable pancreatic cancer; however, only 20% pancreatic cancer patients are eligible for radical surgery (9). The 5-year survival rate of pancreatic cancer patients after surgery is 12%–27% (10); in advanced pancreatic cancer, the 5-year survival rate is <7% (11). Esophageal cancer ranks seventh and sixth in terms of incidence and overall mortality, respectively (4, 12, 13). The treatment approach includes surgery, chemotherapy, and radiotherapy as the mainstay of treatment for advanced esophageal cancer (14). Nevertheless, the prognosis of esophageal cancer is poor, and overall survival (OS) at 5 years is <20% (15). Survival of stomach cancer patients also remain poor (4). The incidence rate is highest in Eastern Asia. Complete resection (R0) is selected for resectable gastric cancer. However, the survival rate of stomach cancer is lower, and the 5-year survival rate is approximately 30%–35% (16). The median survival time is approximately 1 year in advanced gastric cancer patients (17). Primary liver cancer is the sixth most common cancer and the third most lethal tumor (4). The survival of hepatocellular carcinoma (HCC) at 5 years is only 18% (18). The dismay survival is due to the fact that 70%–80% of patients are diagnosed at an advanced stage (19).

Despite recent advances, the prognosis of DSCs remains unsatisfactory (20). Generally, the pathological tumor nodal metastasis (TNM) stage reflects the prognosis in different cancer (21). However, patients with the same stage may have different prognoses. Other markers such as circulating tumor DNA number of mutations have also been used to predict DSC prognosis (22). Hence, identification of valuable markers to guide clinical treatment is urgently needed.

In the tumor microenvironment, cancer immunity plays a vital role in promoting cancer cell proliferation, survival, and angiogenesis (23). In the last decade, immunotherapy has become an important treatment for cancer, and programmed cell death protein 1 (PD-1)/programmed death ligand-1 (PD-L1) are vital pathways (24). The level of PD-L1 in tumor tissues is the most effective biomarker for evaluating patients receiving immunotherapy (25). However, there are limitations that cannot be monitored during treatment, such as dynamic changes of PD-L1, which changes dynamically. PD-L1 is also called CD274 and B7-H1 (26). The soluble forms of PD-1 and PD-L1 were called soluble PD-1 (sPD-1) and soluble PD-L1 (sPD-L1), respectively (25, 27). sPD-L1 is expressed in both tumors and dendritic cells (27). sPD-L1 may be formed via the proteolytic cleavage of the extracellular portion of the membrane that binds to PD-L1 (28). sPD-L1 retains the ability to inhibit T-cell activation and proliferation (29). Moreover, activation of the PD-1/PD-L1 pathway is associated with tumor evasion, cancer development, and progression (25, 30). Normal human serum can secret sPD-L1, and the levels of sPD-L1 in human serum increases with age (27). Membrane-bound PD-L1 is a prognostic factor in several types of cancer (31). Moreover, some studies have reported that sPD-L1 can be detected in the blood of patients with cancer and is regarded as a prognostic marker (32–35). It was reported that in patients with pancreatic cancer receiving chemotherapy who achieved an objective response, sPD-L1 levels were significantly higher with disease progression. In addition, dynamic changes in sPD-L1 levels during treatment are associated with disease progression (36). Nonetheless, the prognostic value of sPD-L1 expression in cancer remains controversial (25). Several meta-analyses have been carried out to investigated the predictive role of sPD-L1 in non-small cell lung cancer (35) and solid tumors (24). There was no previous meta-analysis focusing on this topic in DSCs. Therefore, a meta-analysis was carried out to determine the prognostic value of sPD-L1 in DSCs.

## 2 Methods

### 2.1 Literature search

The Preferred Reporting Items for Systematic Reviews and Meta-Analyses (PRISMA) guidelines were used in this study (37). PubMed, Web of Science, EMBASE, and the Cochrane Library electronic databases were searched. The search used the following MeSH terms and keywords: cancer, carcinoma, tumor, or neoplasm; soluble programmed cell death-ligand 1 (sPD-L1) or programmed cell death-1 or (PD-1) or PD-l1; and survival, predictive, prognosis, or prognostic. The deadline for the search was 1 February 2021. Additional searches were conducted to screen the references of the included studies for potentially missing studies that met the inclusion criteria. Two independent researchers conducted this study.

### 2.2 Inclusion and exclusion criteria for the meta-analysis

The inclusion criteria were as follows: a) patients diagnosed with malignant DSCs (such as pancreatic cancer, colorectal cancer, liver cancer, gastric cancer, esophageal cancer, and biliary tract cancer) confirmed by pathological analysis; b) the studies were conducted in English; c) human survival ([OS] or disease-free survival [DFS]) with regard to sPD-L1 levels is provided by hazard ratios (HRs) or survival curves or can be calculated from the text; and d) each study had a sample size of more than 20 cases. The exclusion criteria were as follows: a) letters to the editor, comments, reviews, and animal studies; b) the sample sizes were <20 for each cancer type; and c) survival data were not provided.

### 2.3 Data extraction

Data were extracted by two independent reviewers. Information from the included studies was reviewed and extracted. This included the following:

1) Authors, publication years, countries, histological types (differentiation), gender, tumor stage, metastases stage, initial treatment methods (surgery, chemotherapy, or radiotherapy), study types (retrospective or prospective), sample sizes, ages, the methods for sPD-L1 detection, the cutoff value of sPD-L1, and follow-up time.
2) OS and DFS and the predictive value of sPD-L1 for treatment response and metastasis.

### 2.4 Quality assessment

The Newcastle–Ottawa Quality Assessment Scale (NOS) was used to evaluate study quality, as previously described (38). The scores ranged from 0 to 9 according to the quality of the studies. A score equal to or higher than 6 was regarded as high quality. The quality assessment was performed by two independent reviewers. Any disagreements regarding the study selection, data extraction, and quality assessment were resolved by a third reviewer.

### 2.5 Statistical analysis

Review Manage (5.3 version) software (Nordic Cochrane Centre) and STATA software (version 12.0) were used for data evaluation (39). The correlation between sPD-L1 expression and survival outcomes were recorded using HRs and 95% confidence intervals (CI) (39). We used the χ^2^ and I^2^ tests to quantify the heterogeneity (39). Heterogeneity was evaluated using I^2^, and the values of 25%, 50%, and 75% were considered low, moderate, and high, respectively (40). If I^2^ <2.5%, data analysis was performed using a fixed-effects model. Otherwise, a random effects model was used. Statistical significance was set at p <0.05. Subgroup and sensitivity analyses were performed. Sensitivity analysis is an important method to evaluate the robustness and reliability of combined results in meta-analysis. Publication bias was evaluated using the Begg’s test (41).

## 3 Results

**Figure 1:**
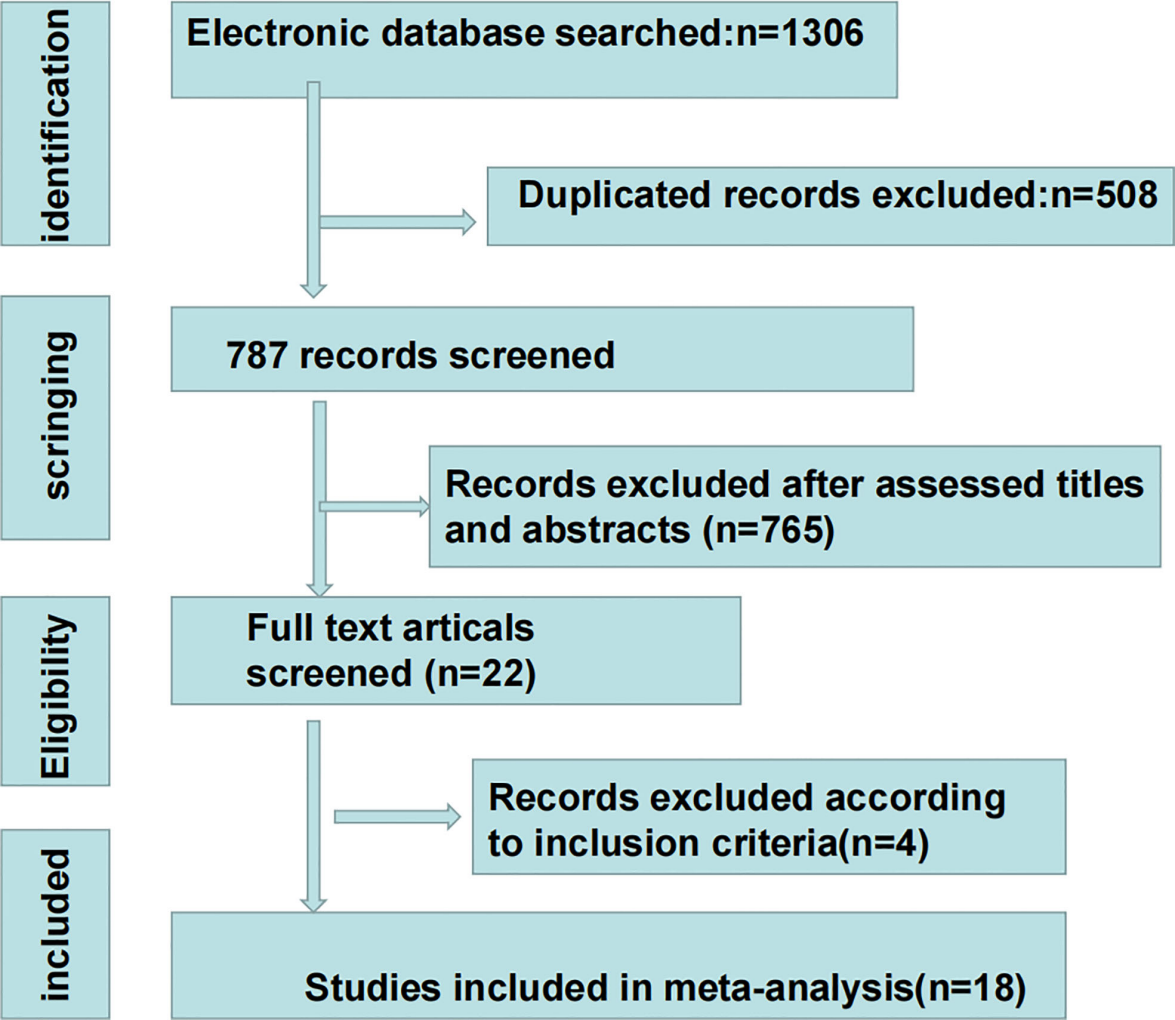
The flowchart of study selection.

The selection flowchart is shown in **Figure 1**. A total of 223, 464, 608, and 11 studies were identified from PubMed, Web of Science, EMBASE, and the Cochrane Library, respectively. Duplicate references (n=508) were removed using Note-express software. After screening the titles and abstracts, 22 papers were required for full-text screening. One study was excluded due to inclusion of fewer than 20 patients (42). Other studies were excluded owing to a lack of relevant survival outcome data (43–45). In all, 18 studies involving 2,070 patients met the inclusion criteria, with six studies focusing on gastric cancer (46–51), six on HCC (52–57), three on pancreatic cancer (36, 58, 59), one on biliary tract cancer (60), one on rectal cancer (61), and one on esophageal carcinoma (62). Sample sizes ranged from 25 to 313. The years of publication ranged from 2016 to 2021. The basic information of including studies and NOS scale are listed in **Table 1**. The sPD-L1 was detected using enzyme-linked immunosorbent assay (ELISA) in all the studies. OS was described in 17 studies, and DFS was mentioned in 10 studies. The median OS in the high sPD-L1 group and the prognostic role of sPD-L1 in terms of OS, treatment response, and metastases are summarized in **Table 2**.

Table 1The information of included studies and quality assessment.

**Table:** 

Study	Country	Sample sizes	ages	Cancer types	Methods detection	Cutoff value	Outcomes	Follow-up time	Quality score
Bian 2019	France	32	65(48–84)	PC	Elisa	>0.36ng/ml	OS	6.9(4.4–10.19	7
Chang 2019	China	120	NA	HCC	Elisa	11.2μg/ml	OS,DFS	NA	6
EI-Gebaly 2019	Egypt	25	59.24 ± 7.67	HCC	Elisa	NA	OS	24M	7
Fan 2019	China	69	64(42–81)	GC	Elisa	82.585ng/ml	OS	26.9(0.8–51.2)	7
Finkmelmeier 2016	Germany	215	64(38–86)	HCC	Elisa	>0.8ng/ml	OS	298–304 days	7
Fu 2021	China	190	NA	EC	Elisa	>0.63ng/ml	OS	NA	7
Ha 2016	Korea	158	59.6(31.3–76.2)	BTC	Elisa	>0.94ng/ml	OS	95.3	8
Han 2018	China	81	NA	HCC	Elisa	2.825ng/ml	OS,DFS	NA	8
Ito 2018	Japan	152	69.9(35–93)	GC	Elisa	≥50μg/ml	OS,DFS	NA	7
Kim 2018	Korea	53	NA	HCC	Elisa	≥1.315pg/ml	OS	21.3(1.7–68.4)m	6
Kruger 2017	Germany	41	NA	PC	Elisa	0.117ng/ml	OS	24.7(19.6–30)	7
Li 2021	China	313	NA	GC	Elisa	≥8.75pg/ml	OS,DFS	18M	8
Mocan 2021	Romania	121	NA	HCC	Elisa	96pg/ml	OS,DFS	24	8
Park 2019	Korea	60	NA	PC	Elisa	≥4.6μg/ml	OS,DFS	11.4(6.9–14.8)	7
Park 2021	Korea	68	56(28–80)	GC	Elisa	1.92ng/ml	OS,DFS	44.3(41–NA)	8
Shigemori 2018	Japan	180	NA	GC	Elisa	>0.507ng/ml	OS,DFS	NA	8
Takahashi 2016	Japan	75	67(39–79)	GC	Elisa	0.704ng/mL	OS,DFS	NA	7
Tominaga 2019	Japan	117	61(27–79)	RC	Elisa	0.156ng/ml	DFS	33.7(8.0–60.6)	7

**Table:** 

Study	Histological type (well+moderate/poor)		Ages	Sex ratio (M/F)	M stage (M0/m1)	Tumor stage (T1+T2/T3+T4)	Study type	Initial treatment	
Bian 2019	NA		65(48–84)	17/15	12/20	NA	retrospective	NA	
Chang 2019	NA		NA	105/15	120/0	NA	retrospective	surgery	
EI-Gebaly 2019	NA		59.24 ± 7.67	NA	NA	NA	prospective	NA	
Fan 2019	18/51		64(42–81)	43/26	69/0	12/57	retrospective	surgery	
Finkmelmeier 2016	NA		64(38–86)	171/44	202/13	NA	prospective	NA	
Fu 2021	98/92		NA	87/103	110/80	35/155	retrospective	chemotherapy	
Ha 2016	NA		59.6(31.3–76.2)	103/55	NA	NA	retrospective	chemotherapy	
Han 2018	NA		NA	75/6	NA	61/20	retrospective	surgery/ablation	
Ito 2018	79/73		69.9(35–93)	103/49	130/22	NA	retrospective	surgery	
Kim 2018	NA		NA	42/11	53/0	29/24	prospective	radiotherapy	
Kruger 2017	NA		NA	NA	6/35	NA	prospective	chemotherapy	
Li 2021	NA		NA	219/94	280/33	121/192	retrospective	surgery	
Mocan 2021	NA		NA	83/38	83/38	NA	prospective	surgery	
Park 2019	NA		NA	33/27	14/46	NA	retrospective	chemotherapy	
Park 2021	30/38		56(28–80)	43/25	NA	1/116	prospective	chemotherapy	
Shigemori 2018	78/102		NA	116/64	165/15	108/72	retrospective	surgery	
Takahashi 2016	34/41		67(39–79)	58/17	0/72	NA	retrospective	chemotherapy	
Tominaga 2019	113/4		61(27–79)	77/40	117/0	1/116	prospective	chemoradiotherapy	

BTC, biliary tract cancer; DFS, disease-free survival; EC, esophageal carcinoma; GC, gastric cancer; HCC, hepatic cell carcinoma; NA, not available; OS, overall survival; PC, pancreatic cancer; RC, rectal cancer.

**Table 2:** Studies on clinical significance of sPD-L1 in malignant digestive system cancer.

Study	Cancer type	Median OS (month) in higher level of sPD-L1 group	Outcomes (prognostic value in OS, DFS, metastasis/recurrence, treatment response, and the association with clinical features)
Bian 2019	PC	Learning cohort: 2.8 Validation cohort: 9.41	sPD-L1 levels negatively correlate with OS.
Chang 2018	HCC	NA	1. sPD-L1 was a negative independent prognostic factor (DFS: HR: 2.58, p=0.023; OS: HR, 1.77, p=0.048). 2. sPD-L1 positively correlated with inflammatory cytokines. 3. sPD-L1 was not related with BCLC stage.
EI-Gebaly 2019	HCC	4.17	sPD-L1 increased risk of mortality.
Fan 2019	GC	NA	1. sPD-L1 was not correlation with OS. 2. sPD-L1 was associated with the age and location of GC
Finkelmeier 2016	HCC	NA	1. High sPD-L1 level had an increased mortality risk (HR, 3.340, p < 0.001), 2. sPD-L1 level was related with BCLC staging system.
Fu 2021	EC	13	1. sPD-L1 level and tissue PD-L1 expression level was not significant correlation. 2. Patients with high sPD-L1 level (≥0.63 ng/ml) was associated with shorter OS than those patients with a low sPD-L1 level.
Ha 2016	BTC	7.93	sPDL1 was negatively related with OS (HR, 1.891, p<0.001).
Han 2018	HCC	5.6	1. sPD-L1 levels were related with DFS (HR, 3.503; p=0.002) and OS (HR, 3.399, p=0.012). 2. sPD-L1 level was positive correlation with tumor PD-L1 expression.
Ito 2018	GC	NA	1. sPD-l1 was related with age but not related to stage. 2. sPD-L1 was associated with RFS and OS.
Kim 2018	HCC	NA	1. Initial sPD-L1 level was significantly related with stage and tumor size. 2. Patients with a higher level of sPD-L1 at 1 month (12.9 pg/ml) showed poorer lung metastasis-free survival. 3. Higher level of sPD-L1 was related with poorer OS.
Kruger 2017	PC	11.92	1. No correlation of sPD-L1 levels with tumoral PD-L1 expression was found. 2. sPD-L1 level was not associated with OS (11.92 vs. 9.53 month for high sPD-L1 vs. low sPD-L1, p=0.36)
Li 2021	GC	NA	1. High sPD-L1 level was related to TNM stage and metastases. 2. There was no relation between OS and sPD-L1 level. 3. High preoperative sPD-L1 level was related with worse RFS.
Mocan 2021	HCC	NA	1. sPD-L1 level was related with DFS (HR, 5.42; p<0.001) and OS (HR, 9.67; p<0.001). 2. High sPD-L1 predict high level of recurrence (p<0.01); there was no relation to complete treatment 3. A positive correlation between sPD-L1 and PD-L1 expression in cancer cells was found (p=0.01).
Park 2021	GC	9.5	1. Patients with low levels of sPDL1 at diagnosis (<1.92 ng/ml) showed a better OS and PFS than patients with a high sPD-L1. 2. sPDL1 value increased after progression compared with baseline in the PR group and the SD group, the level of sPD-L1 was increased with disease progression.
Shigemori 2018	GC	NA	1. sPD-L1 was not correlated with any clinicopathological factors. 2. High sPD-L1 was associated with poorer OS and DFS. 3. sPD-L1 level was a predictor factor for recurrence, but not related to metastases
Takaha 2018	GC	13.2	1. There was no significant difference in sPD-L1 levels according to the response to first-line treatment. 2. High sPD-L1 was more frequently observed in patients with high levels of sPD-L1. The frequency of subsequent treatment after failure of first-line treatment was not different between the high and low sPD-L1 groups. 3. High sPD-L1 levels were associated with worse OS (HR, 2.218, p= 0.01).
Park 2019	PC	8	1. sPD-L1 at diagnosis could not predict the ORR, a decreased sPD-L1 level at the first response could predict ORR. 2. sPD-L1 level (<4.6ng/ml) at diagnosis was a negatively prognostic factor for OS (p=0.015).
Tominaga 2019	RC	NA	1. Remission was 11 (12.7%) in low sPD-L1 group, 2 (6.7%) in high sPD-L1 group. 2. High sPD-L1 level after CRT tended to be associated with worse DFS. 3. sPD-L1 level was not related with tissue PD-L1 expression. 4. Baseline sPD-L1 was significantly associated with younger age (p = 0.044), and high sPD-L1 after treatment was significantly associated with lymphovascular invasion (p = 0.021).

OS, overall survival; DFS. disease-free survival; BCLC, Barcelona Clinic Liver Cancer; CRT, chemoradiotherapy; ORR, objective response rate; BTC, biliary tract cancer; EC, esophageal carcinoma; GC, gastric cancer; HCC, hepatic cell carcinoma; NA, not available; PC, pancreatic cancer; RC, rectal cancer.

### 3.1 High sPD-L1 level and survival outcomes in DSCs

**Figure 2:**
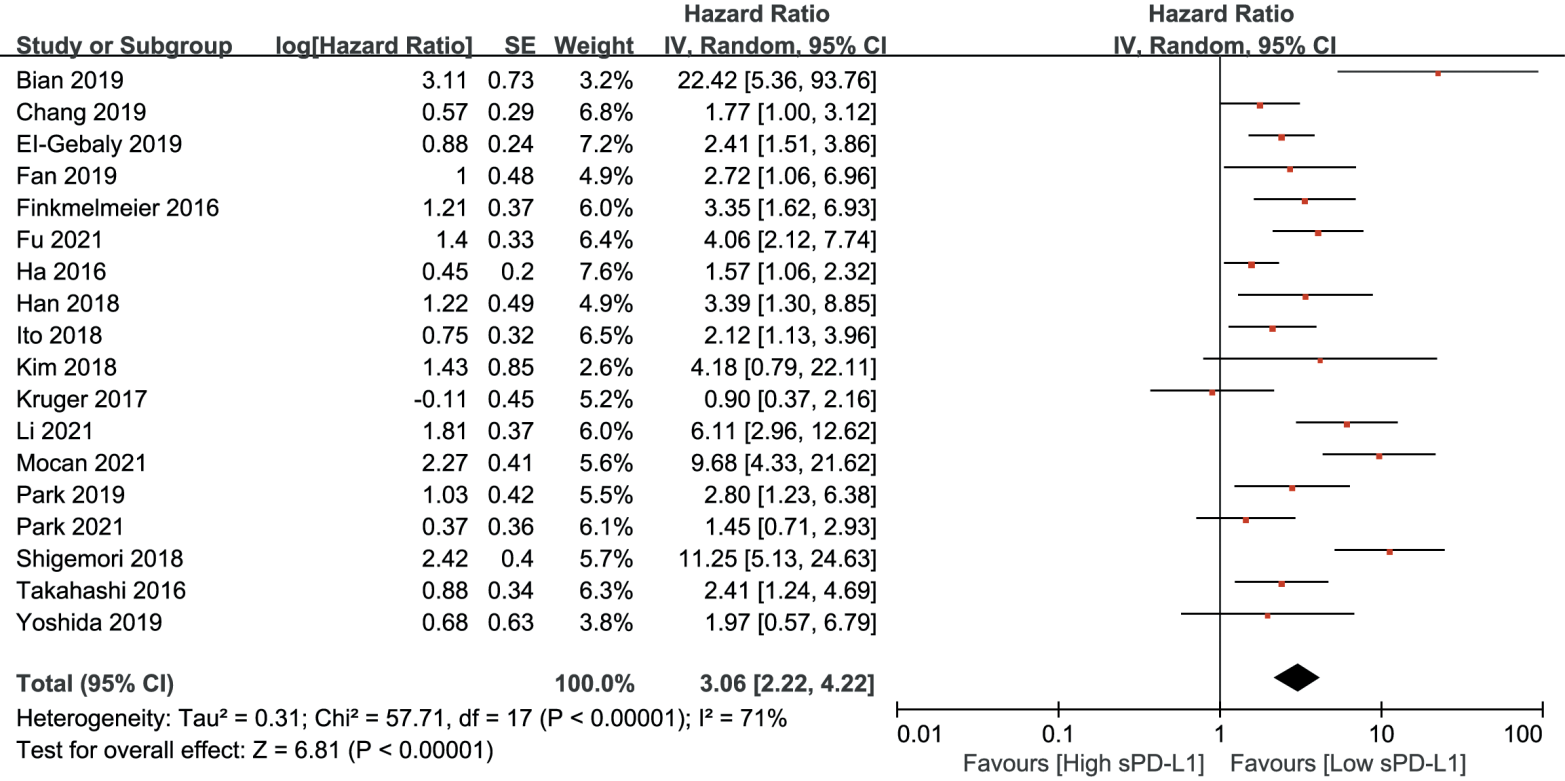
Forest plot of hazard ratio (HR) for the relationship between sPD-L1 level and overall survival (OS).

**Figure 3:**
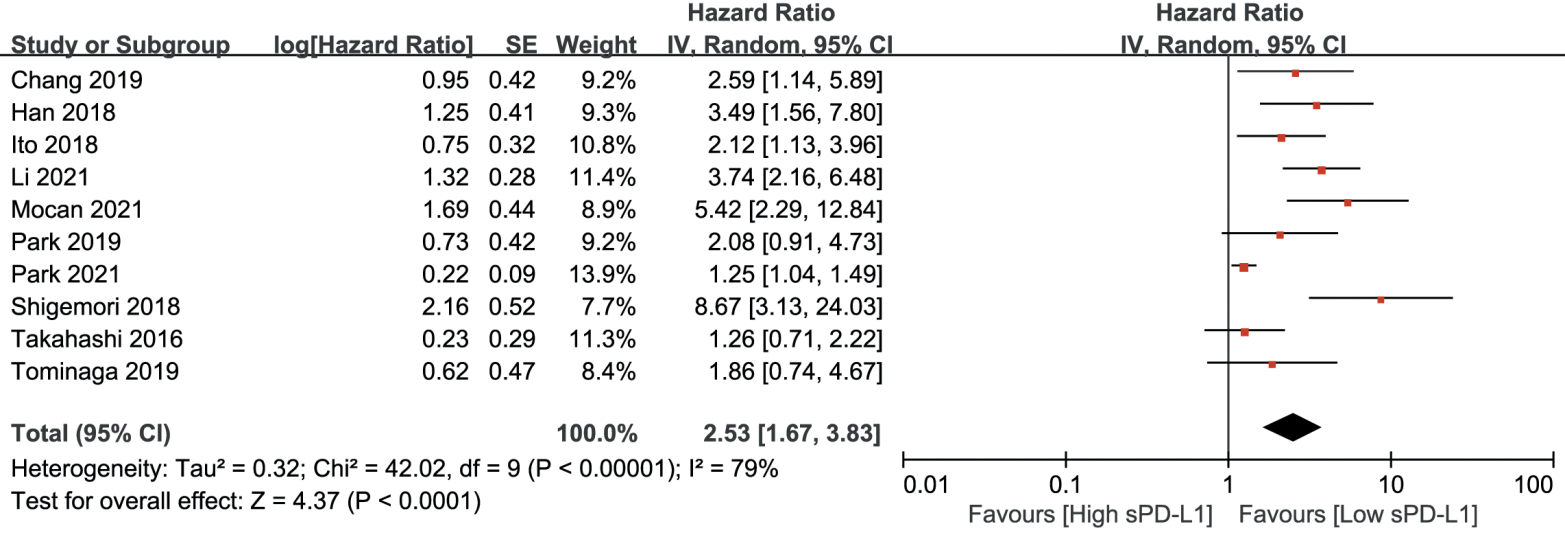
Forest plot of hazard ratio (HR) for the relationship between sPD-L1 level and disease-free survival (DFS).

As shown in **Figure 2**, an HR of 3.06 (95% CI, 2.22–4.22, p<0.001) indicated that a higher sPD-L1 level predicted worse OS in the pooled data of 18 studies. A random-effects model was applied owing to the high heterogeneity among the studies (I^2 = ^71, p<0.001). Furthermore, a high sPD-L1 level was correlated with unfavorable DFS by pooling the data from 10 studies using a random-effects model (HR, 2.53; 95% CI: 1.67–3.83); p-value was <0.01 with significant heterogeneity (I^2 = ^79, p<0.001) (**Figure 3**).

### 3.2 sPD-L1 level and survival in HCC

Six studies focused on sPD-L1 levels and survival outcomes in HCC (52–57). A study by Mocan et al. (57), which included 121 patients with HCC, identified that the best cutoff value of sPD-L1 for both DFS and OS was 96 pg/ml. Patients with high sPD-L1 levels had a shorter DFS (HR, 5.42; p<0.001) and OS (HR, 9.67; p<0.001). The study of Kim et al. (56), which included 53 HCC patients, showed that high sPD-L1 level was associated with poor OS and early lung metastasis but failed to predict local failure-free or progression-free survival (PFS). A study by Han et al. (55), comprising 81 patients with hepatitis B virus-related HCC, suggested that higher sPD-L1 levels were associated with poorer OS (HR, 3.399; p=0.012) and DFS (HR, 3.503; p=0.002). Chang et al. (52) found that sPD-L1 expression was a negative predictive factor for DFS (HR, 2.58; p=0.023) and OS (HR, 1.77; p=0.048) in 120 patients with HCC. El-Gebaly et al. (53) reported that sPD-L1 was an independent prognostic factor for OS in HCC (HR, 2.397; p<0.001) on multivariable analysis. Finkelmeier et al. (54) designed a study to assess the sPD-L1 level and OS in 215 HCC patients. They found that sPD-L1 levels correlated with the Barcelona Clinic Liver Cancer staging system. They also found that high sPD-L1 levels were associated with mortality risk (HR, 3.340; p<0.001). The pooled data of these six studies indicated that a higher level of sPD-L1 was correlated with a poorer OS (HR, 3.28; 95% CI, 2.01–5.35, p<0.001).

### 3.3 sPD-L1 level and survival in gastric cancer

Six studies reported sPD-L1 levels and survival outcomes in gastric cancer patients (46–51). A prospective study (49) from Korea, which included 68 patients with gastric cancer, demonstrated that a high level of sPD-L1 level at diagnosis was correlated with a poorer OS (OS, 9.5 vs. 18.3 months, p=0.057) and PFS (8.9 vs. 6.0 months, p=0.040). Li et al. (48) designed a study to assess the prognostic value of sPD-L1 in 313 patients with gastric cancer. They indicated that postoperative sPD-L1 changes correlated with poor OS (HR, 1.029; p=0.018) and recurrence-free survival (RFS) (HR, 1.029; p=0.011). Ito et al. (47) reported that in 152 patients with gastric cancer, a median sPD-L1 level of 50 pg/ml was the cutoff value and showed that a high sPD-L1 level was associated with poor OS (HR, 2.12; p=0.02). Shigemori et al. (50) designed a study that evaluated the prognostic value of sPD-L1 and tissue PD-L1 in 180 patients with gastric cancer who underwent radical surgery. They found that both tissue PD-L1 and sPD-L1 levels were associated with poorer OS (tissue PD-L1: HR, 4.28; p=0.0094; sPD-L1: HR, 11.2; p=0.0001) and poor DFS (tissue PD-L1: HR, 6.96; p=0.0002; sPD-L1: HR, 8.7; p<0.001). Takahashi et al. (51) included 75 patients with metastatic gastric cancer and found that sPD-L1 level was an independent prognostic factor for gastric cancer (optimal cutoff value: HR, 3.307; p=0.0046; median cut-off value: HR, 2.218; p=0.019). Pooled data of the six studies indicated that high level of sPD-L1 was associated with worse survival (HR, 3.55; 95% CI: 2.01–6.28, p<0.01).

### 3.4 sPD-L1 level and survival in pancreatic cancer

A study (58) from France included 32 patients with pancreatic adenocarcinoma and showed that a high level of sPD-L1 (>0.36 ng/ml) was related with worse OS (median OS, 9.41 months in high level of sPD-L1 vs. 19.87 months in low level of sPD-L1). Kruger et al. (59) showed that sPD-L1 levels are not associated with OS in either univariate or multivariate analyses. Park et al. (36) prospectively included 60 patients with pancreatic cancer and indicated that, by multivariate analysis, patients with high levels of sPD-L1 had worse OS compared to those patients with low levels of sPD-L1 (HR, 3.249; p=0.012; median OS, 8.4 vs. 10.2 months).

### 3.5 sPD-L1 level and survival in esophageal carcinoma

A study from China (62) including 190 patients with esophageal carcinoma indicated that sPD-L1 was highly expressed in female patients with esophageal carcinoma. High sPD-L1 concentrations (≥0.63 ng/ml) were related with a shorter OS (HR, 3.71; p<0.001).

### 3.6 sPD-L1 level and survival in biliary tract cancer

Ha et al. (60) reported on 158 patients with biliary tract cancer and measured their sPD-L1 levels. The median value of sPD-L1 was 1.20 ng/ml and patients with high concentrations of sPD-L1 (≥0.94 ng/ml) were correlated with a poorer OS than patients with low sPD-L1 (HR, 1.89; 95% CI: 1.35–2.65, *p*<0.01).

### 3.7 sPD-L1 level and survival in rectal cancer

sPD-L1 was measured using ELISA before and after neoadjuvant chemoradiotherapy in 117 patients with rectal cancer in a study from the UK (61), which indicated that after neoadjuvant chemoradiotherapy, sPD-L1 levels significantly increased and high sPD-L1 levels before neoadjuvant chemoradiotherapy were related to younger age. High sPD-L1 levels after neoadjuvant chemoradiotherapy were associated with lymphovascular invasion and poor DFS.

### 3.8 Sensitivity analysis and subgroup analysis

**Figure 4:**
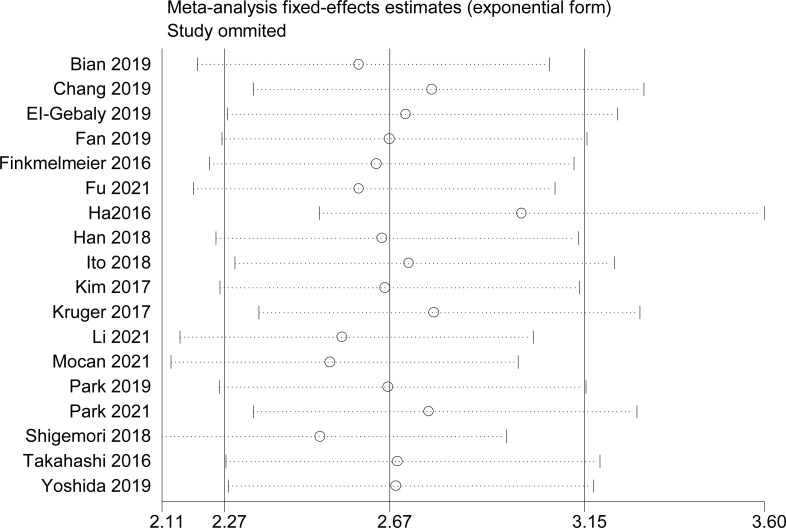
Sensitivity analysis by omitted every single study.

To confirm the stability of the findings, a sensitivity analysis was performed by omitting any single study on OS. The results are reliable, as shown in **Figure 4**. To determine the reliability of the results, subgroup analyses were conducted based on country location, ages, sex, study types, initial treatment, metastases stage, year of publication, sample size, cancer type, and NOS score.

The results are summarized in **Table 3**. High sPD-L1 levels were associated with worse OS in all subgroup analyses except in gender data not provided, indicating reliability of the results.

**Table 3:** Subgroup analyses assessing high sPD-L1 level and overall survival in patients with digestive system cancers.

Items	No.of studies	HR and its 95% CI	Z- value	p-value	Heterogeneity	
					I^2^(%)	p
East country	12	2.88(2.02,4.10)	5.86	<0.001	66	<0.001
West country	5	3.93(1.67,9.24)	2.48	0.002	83	<0.001
Publish year ≥2019	9	3.56(2.25,5.63)	5.43	<0.001	72	<0.001
Publish year <2019	8	2.68(1.62,4.42)	3.84	<0.001	73	<0.001
Sample sizes ≥100	8	3.72(2.20,6.30)	4.91	<0.001	82	<0.001
Sample sizes <100	9	2.53(1.67,3.83)	4.39	<0.001	54	0.03
Quality score≥7	15	3.24(2.25,4.65)	6.3	<0.001	75	<0.001
Quality score<6	2	1.93(1.11,3.31)	2.40	0.02	0	0.34
Gender data provided	15	3.45(2.40,3.97)	6.65	<0.001	72	<0.001
Gender data not provided	2	3.17(0.61,4.13)	0.94	0.35	73	0.05
Mean ages ≥60 years	5	3.26(1.87,5.67)	4.17	<0.001	57	0.05
Mean ages<60 years	3	1.80(1.33,2.44)	3.79	<0.001	14	0.31
Without metastasis	3	2.10(1.32,3.36)	3.12	0.002	0	0.52
Existed metastases	10	4.11(2.51,6.71)	5.64	<0.001	75	<0.001
Retrospective	11	3.40(2.24,5.15)	5.77	<0.001	74	<0.001
Prospective	6	2.65(1.42,4.94)	3.05	0.002	74	0.002
Surgery	7	4.13(2.31,7.37)	4.79	<0.001	76	<0.001
Chemotherapy/radiotherapy	8	2.58(1.53,4.34)	3.55	<0.001	76	<0.001

**Figure 5:**
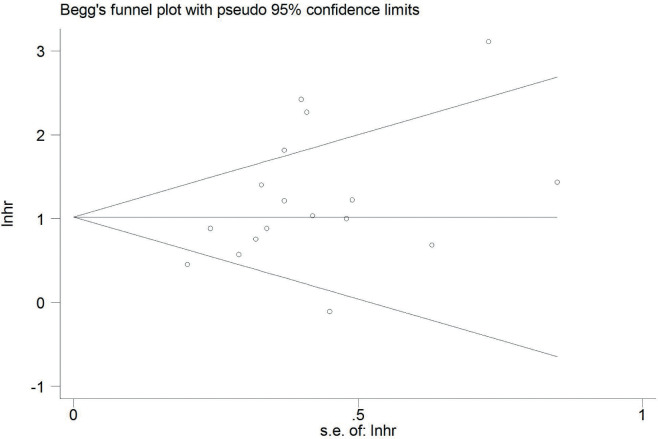
Publication bias evaluated by Begg’s test.

Publication bias was evaluated using Begg’s test for OS. The results are shown in **Figure 5** (p=0.07, Begg’s test). No significant publication bias was observed.

## 4 Discussion

PD-L1 can be divided into membrane-bound PD-L1 and sPD-L1 (25). The detection of sPD-L1 in the plasma of patients with cancer has attracted great interest from researchers. Moreover, some reports indicated that sPD-L1 may be a prognostic factor in cancers (34, 35, 63–66). However, the predictive role of sPD-L1 in DSCs remains controversial.

The results of this meta-analysis revealed that high levels of sPD-L1 were associated with unfavorable OS. Several studies have reported that high sPD-L1 expression is associated with poor survival in breast cancer (25), renal cell carcinoma (63), and other solid cancers (25). However, its predictive role in digestive system cancers has not yet been fully established. Recently, the prognostic role of sPD-L1 in DSCs has been reported. Yoshida et al. reported that sPD-L1 levels are not related to OS (45). In contrast, a study reported by Fu et al. indicated that a high level of sPD-L1 predicted a worse survival outcome (62). This inconsistency requires further investigation. In pancreatic cancer, two studies indicated that higher sPD-L1 levels correlated with worse OS (58, 59). However, another study (36) revealed no significant between sPD-L1 level and survival outcomes in patients with pancreatic cancer using multivariable analysis.

Monitoring sPD-L1 levels might be helpful for predicting survival in patients with cancer and subsequently improving treatment efficacy (62). Tominaga et al. reported that the remission rate was higher in the low sPD-L1 group compared with that in the high sPD-L1 group (49). Park et al. showed that in gastric cancer, with disease progression, the sPD-L1 level increased (36). Some studies have also reported the predictive role of sPD-L1 for detecting metastasis. Kim et al. showed that patients with higher levels of sPD-L1 at 1 month (12.9 pg/ml) had poorer lung-metastasis-free survival (43). Mocan et al. indicated that high sPD-L1 predicted recurrence (57). Shigemori et al. discovered that the sPD-L1 level was a predictor of recurrence but was not related to metastases (37). Therefore, the prognostic value of sPD-L1 requires further investigation.

The potential correlation between sPD-L1 and tissue PD-L1 levels was also investigated. In rectal cancer, PD-L1 expression in biopsy specimens is not significantly different from that in serum PD-L1 (58). In gastric cancer, tissue PD-L1 expression does not correlate with sPD-L1 expression (45). Mocan et al. and Han et al. indicated that tissue PD-L1 is related to sPD-L1 in HCC (50, 52). In pancreatic cancer, no relationship has been observed between tissue PD-L1 and sPD-L1 (54). In esophageal cancer, tissue PD-L1 expression does not correlate with sPD-L1 expression (55). There was a significant correlation between sPD-L1 and tumor PD-L1 expression (51). Overall, the relationship between sPD-L1 and the expression of PD-L1 in tissue requires further investigation.

Several studies have reported the association of inflammatory cytokines with sPD-L1 level (47). In esophageal cancer, the researchers indicated that there was no correlation between sPD-L1 and C-reactive protein (CRP) (45). In another study, the investigator indicated that sPD-L1 was related with white cell count, but not correlated with CRP and other inflammatory markers. However, some studies have indicated that sPD-L1 was related with white blood cell and platelet count (49). Masaaki et al. suggested that sPD-L1 was associated with C-reactive protein levels (47). In HCC, Finkelmeier et al. indicated that sPD-L1 positively correlated with CRP (54). The relationship between sPD-L1 and inflammatory factor should be further investigated in different kinds of cancers.

Several studies indicated that sPD-L1 expression was not correlated with age and sex (36, 45, 46, 48, 50, 52, 61). One study indicated that sPD-L1 expression was related with age, but not correlated with gender (60). By contrast, another study revealed that sPD-L1 was associated with gender, but not related with age (47). However, some studies indicated that older age was associated with higher sPD-L1 (28). Furthermore, in gastric cancer, the expression of sPD-L1 was not significant difference in the intestinal type compared to that in the diffuse type (50).

Inhibition of sPD-L1 can result in a function similar to that of anti-PD-1 or anti-PD-L1 monoclonal antibodies, thereby achieving a checkpoint inhibitory effect (25). Some studies have reported that the inhibition of sPD-L1 restricting tumor growth showed a mechanism similar to that in anti-PD-L1 mAb-injected mice (67, 68). Further evaluation is required to establish the predictive ability of sPD-L1 in cancer treatment.

This study has some limitations. First, some of the studies included in this meta-analysis were retrospective studies, and there might have been selection or publication bias because the positive results were more easily published in the journal, whereas the negative results were not. Second, the cutoff values were not uniform, and heterogeneity might exist. Third, a high heterogeneity was observed in some analyses. The source of heterogeneity may be individual patients with different TNM stages and tumor types, sex, ages, study types, treatment methods, country locations, cutoff values, and follow-up times. To identify the sources of the heterogeneity, subgroup analyses were adopted but failed to determine this.

## 5 Conclusions

In conclusion, sPD-L1 can be a prognostic factor for DSCs. High sPD-L1 expression predicted poor OS and DFS. Inflammatory cytokines, treatment approaches, and other factors may affect the expression of sPD-L1. Therefore, the prognostic value of sPD-L1 for recurrence and metastasis should be further investigated. sPD-L1 may be a prognostic factor for treatment response. Well-designed prospective studies with standard assessment methods should be conducted to determine the prognostic value of sPD-L1 in DSCs.

## Author contributions

Design and manuscript drafting: GL, JR, YQ, and ZZ. Editing and proving: GL, F-MK, and RX. All authors read and approved the final manuscript.
